# Controversies on Endocrine and Reproductive Effects of Glyphosate and Glyphosate-Based Herbicides: A Mini-Review

**DOI:** 10.3389/fendo.2021.627210

**Published:** 2021-03-15

**Authors:** Anderson Tadeu de Araújo-Ramos, Marcella Tapias Passoni, Marco Aurélio Romano, Renata Marino Romano, Anderson Joel Martino-Andrade

**Affiliations:** ^1^ Animal Endocrine and Reproductive Physiology Laboratory, Department of Physiology, Sector of Biological Sciences, Federal University of Paraná, Curitiba, Brazil; ^2^ Reproductive Toxicology Laboratory, Department of Pharmacology, Sector of Biological Sciences, Federal University of Paraná, Curitiba, Brazil; ^3^ Department of Medicine, State University of Midwest Paraná, Guarapuava, Brazil

**Keywords:** pesticide, endocrine-disrupting chemicals, reproductive toxicology, toxicity testing, endocrinology

## Abstract

Glyphosate-based herbicides (GBHs) are among the most used pesticides worldwide, presenting high potential for human exposure. Recently, a debate was raised on glyphosate risks to human health due to conflicting views over its potential carcinogenic and endocrine disruptive properties. Results from regulatory guideline studies, reports from Regulatory Agencies, and some literature studies point to a lack of endocrine disrupting properties of the active ingredient glyphosate. On the other hand, many *in vivo* and *in vitro* studies, using different experimental model systems, have demonstrated that GBHs can disrupt certain hormonal signaling pathways with impacts on the hypothalamic-pituitary-gonadal axis and other organ systems. Importantly, several studies showed that technical-grade glyphosate is less toxic than formulated GBHs, indicating that the mixture of the active ingredient and formulants can have cumulative effects on endocrine and reproductive endpoints, which requires special attention from Regulatory Agencies. In this mini-review, we discuss the controversies related to endocrine-disrupting properties of technical-grade glyphosate and GBHs emphasizing the reproductive system and its implications for human health.

## Introduction

Glyphosate, an aminophosphonic derivative of the natural amino acid glycine, has been extensively used as an herbicide since the early 1970s due to its inhibitory effects on the biosynthesis of essential aromatic amino acids in plants. The development of genetically engineered glyphosate-tolerant crops in 1996 has broadened the agricultural applications of glyphosate-based herbicides (GBHs), which became the most widely used herbicides worldwide (1).

Glyphosate and its main breakdown product aminomethylphosphonic acid (AMPA) have been ubiquitously detected in the environment (2). Surprisingly, there are limited human biomonitoring data on glyphosate, but the available studies indicate ubiquitous presence of both glyphosate and AMPA on human urinary samples from the general population (3–5). Also, GBHs contain a number of co-formulants, which can be potentially ingested (e.g., *via* food or water) concurrently with glyphosate and AMPA (3).

The safety of glyphosate has been the focus of significant concern and debate due to its wide presence in the environment and controversies over its toxicity (6). These controversies can be illustrated by the disputable results of a study by Samsel and Seneff (7), which suggested a link between glyphosate and disruption of metabolic pathways in the gut microbiome and associations with several chronic diseases, such as obesity, depression, autism, cancer, and infertility. However, their comment was based on a flawed logic, lacking scientific evidence, as explored in more detail by Mesnage and Antoniou (8). Some recent data also suggest that glyphosate inhibition of the shikimate pathway could potentially alter gut microbial community and microbiome chemical metabolism in rats (9), although others have found very limited effects of glyphosate on the gut microbiome (10).

The endocrine disrupting potential of glyphosate has been in the center of recent debates, because of conflicting data and interpretation (11–13). According to the Endocrine Disruptor Screening Program (EDSP) of the United States Environmental Protection Agency (14) and reports from the European Food Safety Authority (15) there is no sufficient evidence to support endocrine disrupting effects of glyphosate. These conclusions are based on a set of *in vitro* and short-term *in vivo* screening assays designed to investigate interaction of chemicals with estrogen-, androgen-, and thyroid-signaling pathways or interference with steroidogenesis, as well as *in vivo* long-term toxicity studies (apical studies). A comprehensive review of the EDSP data has been recently published (11). On the other hand, several studies have pointed to potential endocrine and reproductive effects of glyphosate and GBHs in different model systems, with results usually indicating higher toxicity of GBHs when compared to the active ingredient alone. The debate over the endocrine-disrupting effects of glyphosate and other pesticides is of importance in the context of chemical regulation as many countries, including European Union states and Brazil adopt a hazard-based approach to endocrine disruptors in plant protection products (16).

Here, we briefly review some of the studies on glyphosate reproductive and endocrine toxicity and discuss some of reasons for the discrepancies found in the literature. We present experimental data related to effects potentially linked to disruption of sex hormones and possible repercussions on reproductive health. Epidemiological data will not be thoroughly addressed in the current review.

## Interaction of Glyphosate and GBHs With Androgenic and Estrogenic Hormone Pathways or Steroidogenesis

Although controversial, some studies suggested that both technical-grade glyphosate and GBHs could exert receptor-mediated estrogenic or androgenic effects. Thongprakaisang and colleagues (17) have demonstrated that environmentally relevant concentrations of glyphosate promoted the transcription activity of estrogen responsive elements (ERE) and induced *in vitro* growth of the T47D hormone-dependent breast cancer cell lineage, but had no response in the hormone-independent MDA-MB231 cells. Also, they showed that the proliferative effect was abrogated after ICI 182780 (an ER inhibitor) exposure, supporting the estrogenic activity hypothesis of glyphosate. Mesnage et al. (18) showed that technical-grade glyphosate has a weak interaction with estrogen receptor-α (ER-α) and suggested that its gene activation is mediated by a non-ligand binding mechanism.

In opposition, Kojima et al. (19) screened androgenic or estrogenic effects of several pesticides and did not find any hormonal activity of technical-grade glyphosate by using the reporter gene assay in CHO-K1 cells. Recently, Tóth and collaborators (20) used bioluminescent yeast estrogen and androgen assays to screen the estrogenic and androgenic activities of technical-grade glyphosate and co-formulants, and 13 GBHs. technical-grade glyphosate and AMPA displayed no significant hormonal activity, while five GBHs formulations had estrogenic activity, one GBH had androgenic activity and three of the tested formulations displayed both androgenic and estrogenic activity.

Additionally, it has been proposed that both technical-grade glyphosate or GBHs have the ability to disrupt steroidogenesis (21). Overall, it seems the GBHs rather than technical-grade glyphosate are able to disrupt the transcription and activity of some components of the steroidogenic machinery, sometimes leading to changes in the hormone production. Pandey & Rudraiah (21) observed several changes in the adrenal gland of adult male Wistar rats exposed to 10 mg/kg bw of Roundup^®^ during two weeks, including a downregulation of mRNA levels of the steroidogenic acute regulatory protein (StAR), as well as reduced total and phosphorylated StAR protein expression. StAR plays a central role in the biosynthesis of steroid hormones, since it is responsible for the transport of cholesterol to the inner mitochondrial membrane (22). In the study by Pandey & Rudraiah (21), the altered expression of StAR was accompanied by accumulation of lipid droplets in the adrenal gland and increased gland weight and as a consequence, reduced corticosterone levels. Also, Roundup^®^ reduced adrenocorticotropic hormone (ACTH) levels and consequently downregulated the adrenal levels of phosphorylated CREB, a transcriptional factor of the StAR gene (21).

The potential effects of GBHs on the production of sex hormones may be also mediated by disruption of hypothalamic-pituitary-peripheric glands axes. Ren et al. (23) showed that both technical-grade glyphosate and Roundup^®^ reduced the expression of *Gnrh* in the hypothalamus of pregnant mice, but surprisingly also induced an increase in luteinizing hormone (LH) expression in the pituitary, as well as an increase in follicle-stimulating hormone (FSH) expression in the technical-grade glyphosate group. Additionally, our research group has demonstrated that Roundup Transorb^®^ can alter gonadotropin expression in rats. Perinatal exposure to this GBH at 50 mg/kg/day, during a critical window for brain sexual differentiation, enhanced the mRNA expression of *Lh* and *Fsh* and increased LH protein expression in the pituitary and serum LH concentrations in the adult male offspring (24). In line with these results, our study also showed increased circulating levels of testosterone which in turn may have also favored the reported increase in serum estradiol (24).

However, there are conflicting data on the effects of technical-grade glyphosate and GBHs on the levels of circulating sex hormones. While our perinatal study found an increase in serum testosterone concentrations in adult rats exposed *in utero* and during early postnatal life to 50 mg/kg/day Roundup Transorb^®^ (24), in pubertal male rats exposed to this same formulation at 5, 50, and 250 mg/kg/day we detected a dose-dependent reduction in circulating levels of testosterone along with delayed puberty onset (25). Decreased testosterone production was also observed in other experimental settings, including reduced testosterone levels in pubertal and adult rats (13) and quails (26) exposed to GBHs and decreased testosterone output in primary cultures of rat Leydig cells exposed to environmental relevant concentrations of both technical-grade glyphosate and glyphosate-based Roundup Bioforce^®^ formulation (27). Some studies also showed an increase (12, 23, 24) or a decrease (28, 29) in estradiol production, as well as an augmentation (30) or a reduction (23, 29) in the levels of progesterone. However, there are studies reporting no changes in testosterone (31–33), estradiol (30, 32, 33), or progesterone (32, 34) production.

These differences could be related to experimental design, windows and duration of exposure, and in particular to the GBH formulation tested. For example, Johansson and colleagues (31) reported that neither Glyfonova^®^ 450 plus (a GBH formulation) nor technical-grade glyphosate altered intratesticular testosterone concentration and both were not able to promote changes in StAR mRNA or protein levels in the testis of Sprague-Dawley rats. But the same authors found that Glyfonova^®^ 450 plus (25 mg/kg by two weeks) upregulated *Cyp11a1* and *Cyp17a1* mRNA, key steroidogenic enzymes in early steps of steroidogenesis. On the other hand, a pioneer study of GBHs toxicity conducted by Walsh et al. (34) using the MA-10 Leydig tumor cell line found that Roundup^®^ but not technical-grade glyphosate reduced progesterone production, increased the StAR transcripts, and reduced the protein levels of this enzyme. They also reported that Roundup did not interfere with CYP11a1 transcription or translation, but reduced its activity. Also, Roundup^®^ reduced mRNA levels of 3β-HSD (responsible for late steps in testosterone production), but induced no changes in protein levels or enzymatic activity.

Another potential steroidogenic target for GBHs is the CYP19a1 (aromatase) enzyme, responsible for the conversion of androgens into estrogens. Richard et al. (35) reported inhibition of aromatase activity and reduced mRNA transcripts following incubation of JEG-3 human choriocarcinoma derived placental cells for 18h with Roundup^®^, but not with technical-grade glyphosate. The Roundup^®^ tested concentrations were approximately 100 times lower than the recommended concentrations for agricultural use. In this same study, both technical-grade glyphosate and Roundup^®^ inhibited aromatase activity in microsomal fractions obtained from human placenta and equine testis, with the Roundup^®^ formulation displaying an inhibitory effect three times greater than technical-grade glyphosate in the two microsomal preparations.

**Figure 1:**
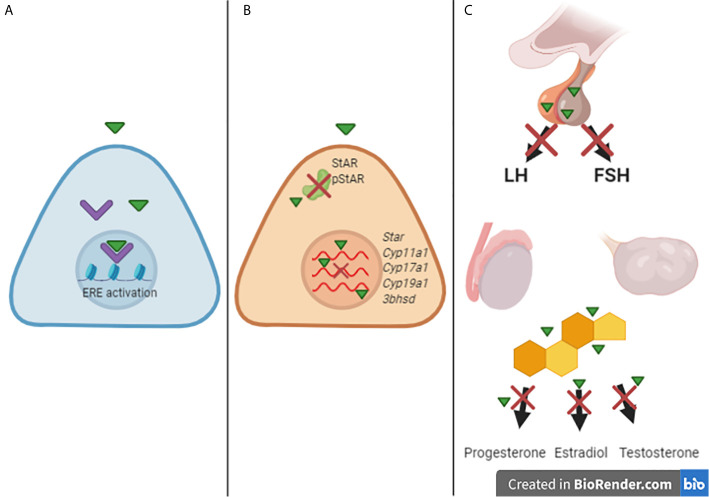
A scheme suggesting some possible targets of endocrine disruption by glyphosate and glyphosate-based herbicides (GBHs). **(A)** Glyphosate/GBH compounds (green triangle) interact with ER-a (purple) and activates the estrogenic-responsive elements transcription. **(B)** glyphosate/GBHs alter the gene expression of steroidogenic machinery and decrease the levels of StAR and phosphorylated StAR. **(C)** glyphosate/GBHs impacts the pituitary homeostasis, changing the production of LH and FSH, as well as in the gonads, impacting steroidogenesis, altering the levels of progesterone, estradiol, and testosterone.

**Figure 1** illustrates some potential sites of interaction of glyphosate/GBHs with components of the endocrine system involved in the control of reproduction. Disruption of steroidogenesis and/or hormonal signaling pathways may be associated with several effects on female and male reproductive function. In **Table 1** we summarize some of the *in vivo* effects observed in experimental animals following technical-grade glyphosate and/or GBHs exposure.

**Table 1:** Summary of the *in vivo* reproductive toxic effects after technical-grade glyphosate or GBH exposure in experimental models.

Chemical	LOAEL for the study	Experimental model	Age at beginning of exposure	Exposure duration	Exposure route	Main results	Reference
Technical-grade glyphosate (Gly) or GBH (Magnum Super, Grupo Agros S.R.L.)	2 mg/kg	Pregnant Wistar rats (F0 dams)	Gestational day 9 until weaning (lactation day 21)	35 days	Oral (laboratory pellet chow based paste)	↑ Food intake (F0 dams; Gly) ↓ ♂ birth weight (Gly) ↑ Preimplantation loss rate (F1 dams; Gly and GBH) ↑ E2 serum levels (F1 pregnant dams; Gly and GBH) ↑ Uterine expression of ER alfa, PR (Gly), Lif, Hoxa10 (Gly and GBH)	Lorenz et al. (12)
Technical-grade glyphosate (Gly) or GBH (Roundup Bioflow, Consorzio Agrario dell’Emilia)	1.75 mg/kg	Pregnant Sprague-Dawley rats (F0 dams) and F1 offspring	F0 dams: Gestational day 9 until weaning (lactation day 21) F1 offspring: until postnatal day 120	F0 dams: 35 days F1 offspring: 134 days	Drinking water	↑Anogenital distance (♂ Gly and GBH; ♀ GBH) Delayed first estrous (GBH) ↑ Epididymis weight (GBH) ↑ Serum DHT ♂ (GBH) ↑ Serum testosterone ♀ (GBH)	Manservisi et al. (13)
GBH (Roundup, Monsanto Co.)	10 mg/kg	♂ Wistar rats	2–2.5 months old	14 days	Gavage	↓ Corticosterone ↓ StAR, CREB, Ldlr, and Hmgcs expression in adrenal gland	Pandey and Rudraiah (21)
Technical-grade glyphosate (Gly) or GBH (Roundup, Sinochem Crop Protection Products Co., Ltd.)	0.5%	Pregnant mice	Gestational day 1 to 19	19 days	Drinking water	↓ Uterus weight (Gly and GBH) ↓ Progesterone (Gly and GBH) ↓ Gnrh mRNA expression (Gly and GBH) ↑ FSH and ↓ FSHR mRNA expression (Gly) ↑ LH mRNA expression (Gly and GBH) and ↑ LHR mRNA expression (GBH) ↑ Cyp11a1 and Cyp19a1 mRNA expression (Gly) ↑ Cyp17a1 mRNA expression (GBH) ↓ 3βHSD mRNA expression (Gly and GBH)	Ren et al. (23)
GBH (Roundup Transorb, Monsanto Co.)	50 mg/kg	♂ Wistar rats (adult male offspring from dams treated with GBH)	Gestational day 18 to postnatal day 5	10 days	Gavage to the mothers	↑ Testosterone, estradiol, LH ↑ LH expression ↑ Sperm production	Romano et al. (24)
Technical-grade glyphosate (Gly)	500 mg/kg	Sprague-Dawley rats	56-day-old	5 weeks	Gavage	↓ Sperm production	Dai et al. (32)
GBH (Roundup Transorb, Monsanto Co.)	5 mg/kg	♂ Wistar rats	23-day-old	30 days	Gavage	↓ Seminiferous epithelium ↓ Testosterone	Romano et al. (25)
Technical-grade glyphosate	0.36 mg/L	Fresh Human Sperm	NA	1 h	Cell culture media	↓ PRM: progressive motility	Anifandis et al. (36)
GBH (RoundupGrand Travaux Plus, Monsanto Co.)	0.5%	♂ Sprague-Dawley (SD) Rats	PND 60	8 days	Drinking water	↑ P450 arom, ↑ Occludin and Connexin 43 expression ↑ Gper1 ↓ Morphology (% normal forms)	Cassault-Meyer et al. (37)
GBH (Roundup Bioflow)	5 μg/mL	Boar semen	2–3 years	1-3 h	Cell culture media	↓ Progressive sperm motility	Nerozzi et al. (38)
Technical-grade glyphosate GLY Sigma-Aldrich	5 mg/kg	♂ Wistar rats	3–4 weeks	52 days	Gavage, ingredients dissolved in corn oil.	↓ Epididymis weights ↓ CAT activity ↓ Plasma testosterone ↓ Sperm counts per epididymides and per testis ↓ Live/dead ratio, % ↑ Total abnormal sperms, % ↓ Sperm motility, %.	Abarikwu et al. (39)
Technical-grade glyphosate GLY Sigma-Aldrich, and Roundup 3 Plus (Monsanto Europe, Belgium)	0.5 mg/kg	Swiss mouse	4 month	E10.5 to 20 dpp	Drinking water	↓ Relative weight Testis ↓ Seminal vesicles weight ↓ Spermatozoa number in epididymis ↓ Serum testosterone levels ↑ Vacuoles in the seminiferous epithelium of the testis	Pham et al. (40)
Roundup Original, Monsanto Co.	50 mg/kg	♂ Wistar rats	23 days	35 days	Gavage	↓ Testosterone levels. ↓ Spermatids/testicle weight ↑ Relative weight of cauda epididymis	Nardi et al. (41)
Roundup Original, Monsanto Co	0.72 ppm	♂ Wistar rats, Sertoli Cells Culture	30 days	72 h	Cell culture media	↑ Oxidative Stress ↑ Ca^++^ uptake	De Liz Oliveira Cavalli et al. (42)
GBH (Roundup Original, Monsanto Co.)	0.5%	C57Bl/6 Mice	Gestational day 4 to postnatal day 21	38 days	Drinking water	↓ BW gain during pregnancy ↑ Age at testis descent ↓ Spermatozoa reserve ↓ Epithelial height of seminiferous tubules ↑ Intratesticular testosterone concentration ↑ LH plasma concentrations	Teleken et al. (43)
GBH (Roundup Full II, Monsanto Co.)	2 mg/kg	♀ Wistar rats offspring	1 – 7 Pnd	7 days	Subcutaneous injection	↑ Sensitivity of the rat uterus to estradiol and Uterine Luminal epithelial height ↑ Stromal thickness ↑ Uterine cell proliferation ↑ ESR1 and ESR 2 expression	Schimpf et al. (44)

LOAEL, lowest observed adverse effect level.

## Examples of Reproductive Effects Linked to Endocrine Modes of Action in Females

Technical-grade glyphosate and its formulations can alter decidualization and pregnancy and it seems that the uterus is extremely sensitive to GBHs. A study showed that neonatal exposure to a GBH containing 66.2% of glyphosate (brand not mentioned) increased the number of reabsorption sites and altered decidualization response in rats, by decreasing the expression of estrogen and progesterone receptors and downregulating COUP-TFII and Bmp2 mRNA (45). Moreover, it was demonstrated later by the same group that GBH neonatal exposure also deregulate the Wnt/β-catenin signaling in the uterus, which might explain embryo losses (46) and enhanced the uterine sensibility to estradiol in ovariectomized rats (44). These data together with possible effects on steroid hormone production (described in the above section) can provide a mechanistic explanation for the reported changes in uterus biology and pregnancy-related problems after GBH exposure, such as disrupted uterine differentiation and miscarriage (34) in animal models, and support the biological plausibility of the association found between glyphosate exposure and shortened pregnancy in humans (47).

Another fact that cannot be ignored is the eminent epigenetic effects of glyphosate exposure. Recently, a study conducted by Kubsad and colleagues (48) demonstrated that exposure of rat F0 dams to 25 mg/kg of technical-grade glyphosate between gestational days 8 and 14 induced reproductive problems such as delayed puberty onset in F2 generation, parturition abnormalities in F3 generation, and ovary diseases in both F2 and F3 generations, indicating direct and transgenerational epigenetic effects.

## Examples of Reproductive Effects Linked to Endocrine Modes of Action in Males

The impact of developmental GBHs exposure on the male reproductive system has been demonstrated in a number of experimental studies. Our research group showed that perinatal rat exposure (GD18 to PND5) to 50 mg/kg Roundup^®^ Transorb disrupted the hypothalamic-pituitary-testis axis and promoted a pro-androgenic effect, by increasing blood levels of testosterone, enhancing the weight of the reproductive organs and sperm production at adulthood (24). This pro-androgenic effect was also recently corroborated by a study by Manservisi et al. (13), who reported increased anogenital distance in rats exposed *in utero* and postnatally to Roundup^®^ Bioflow. However, the disruption of androgen signaling by GBHs seems to be dependent on the timing of exposure and experimental conditions. When administered during the prepubertal period, Roundup^®^ Transorb induced an anti-androgenic response, decreasing the levels of circulating testosterone and delaying the puberty entry in male rats (25). In mice, Roundup^®^ exposure during the gestation and lactation delayed the testis descent and reduced the number of spermatozoa in the cauda epididymis (43). It was demonstrated that immature Sertoli cells are sensitive to GBHs (49) and this seems to be a result of the deregulation of Ca^2+^ homeostasis, triggering the MAPK signaling and reactive oxygen species generation, promoting cell death (42). *In vitro* data indicate disruption of cell detoxification systems by GBHs in testicular cells and usually higher cytotoxicity when compared to technical-grade glyphosate, indicating that co-exposure of the active ingredient and formulants may have greater impact on the testis (34). Also, GBHs may display higher toxicity than some isolated formulants, such as polyethoxylated amine (POEA) compounds used in glyphosate formulations (34). Although GBHs-induced dysfunctions of testicular cells may not be a direct consequence of hormonal disruption, such effects may have significant consequences for testicular function.

The final consequence of the GBH exposure during developmental periods (24, 40, 41, 43) or at adulthood (32, 39) is the potential reduction of male fertility by reducing the sperm quality and count. A recent meta-analysis corroborated the reduction of sperm count due to GBH exposure in rodents (50). Some *in vitro* studies were employed to explore the direct effects of both technical-grade glyphosate and Roundup^®^ in sperm. Nerozzi and colleagues (38) revealed that both Roundup^®^ and technical-grade glyphosate reduced the sperm function, but only Roundup reduced the sperm number. Also, Cassault-Meyer et al. (37) observed that rats exposed to Roundup^®^ showed a decreased sperm nuclear quality and impairment in morphology, although they have not seen any reduction in sperm count and motility. Finally, *in vitro* exposure to 0.36 mg.L^-1^ of technical-grade glyphosate after 1 h reduced the progressive motility but did not affect DNA integrity in human sperm (36).

## Discussion

A central point in the discussion of the endocrine-disrupting potential of glyphosate and GBHs lies in the issue of the toxicological evaluation of technical pesticides or their commercial formulations (3, 51). Currently, standard testing guidelines and regulatory bodies in different countries, including regulatory agencies in the European Union, the United States, and Brazil only require a complete toxicological assessment, including testing of endocrine disruption potential, of active ingredients (16). The literature data on GBHs effects on endocrine sensitive endpoints in different model systems illustrate the limitation of the current approach. GBHs are chemical mixtures and the toxicological assessment of the active ingredient alone is inadequate to predict the toxicity of the commercial formulations (51, 52). In addition, different formulations may result in different toxicity profiles depending on the co-formulants present in each GBH, as discussed in *Interaction of Glyphosate and GBHs with Androgenic and Estrogenic Hormone Pathways or Steroidogenesis*. However, considering the several GBHs mixtures available in the market and that their inert ingredients have not been investigated yet, the regulatory agencies should take into account that not only technical-grade glyphosate but the GBH formulation must be examined for legal decisions and approval. Although we understand the costs of widening the regulatory requirements to commercial formulations, including the increase in the use of laboratory animals, an extended testing strategy for GBHs and other commercial pesticides is needed. This may include the testing of the most widely used formulations as previously proposed (51), but also the use of current and novel *in silico* and *in vitro* methods for the screening of endocrine-disrupting chemicals (EDCs).

Another potential source of disagreement between toxicity studies on endocrine-related effects of active ingredients of pesticides and their commercial formulations may be linked to the limitations of the current regulatory testing strategies (16, 51). Even the complete set of required tests for active ingredients exhibit potential data gaps in the assessment of endocrine-disrupting mechanisms and associated apical effects. For instance, the current regulatory screening assays (e.g., Tier 1 of US EPA) are limited to a number of mechanisms related to steroidogenesis or androgenic-, estrogenic-, and thyroid-hormone pathways and do not take into account possible interactions with additional nuclear and membrane receptors or other receptor-independent mechanisms, such as expression of hormones receptors, hormone transport, and epigenetic alterations (16). Although regulatory apical studies, such as the extended one-generation reproductive toxicity study (OECD guideline 443), are designed to investigate sensitive life stages and a number of endocrine-, immune-, and neurodevelopmental endpoints, there are still important weaknesses concerning endocrine-disrupting effects linked to early-life programming events, such as hormonal imprinting during brain development, neurogenesis, gene expression in hormone-sensitive brain areas, and associated behavioral changes (53). Current testing strategies also fail to consider some aspects of metabolic disruption and effects on hormonal systems associated with stress responses, obesity, and energetic metabolism. It is important to mention that some of the neurodevelopmental and metabolic endpoints mentioned here have been at least partially investigated in prior glyphosate studies, but are outside of the main focus of this mini-review (54–56). Also, many studies ignore the effects of chemicals on females and may, therefore, miss important information on the toxicity profile of EDCs, which can alter hormone driven sex-specific processes at different life stages (57).

In a general way, differences between toxicity studies on glyphosate and other chemicals may arise from the variability in experimental conditions, including laboratory settings, animal species/strain, sex, duration of exposure, and life stage of treatment and analyses. Overall, the debate over the endocrine disrupting properties of technical-grade glyphosate and GBHs illustrates important issues regarding the toxicity testing and regulation of pesticides and other environmental chemicals. In particular, (i) the need to consider exposure scenarios that are human and environmentally relevant, such as testing pesticides formulations and other chemicals mixtures present in real life as opposed to isolated compounds; (ii) a critical appraisal and review of current toxicity testing strategies on EDCs to include a wider range of screening assays designed to detect a larger number of endocrine-related mechanisms, as well as the inclusion of toxicity endpoints that take into account subtle effects induced during early-life programming events. Importantly, the safety assessment limitations underlined here are not limited to glyphosate but could be extended to other chemicals and products of anthropogenic origin.

## Author Contributions

All authors (AA-R, MP, MR, RR, and AM-A) contributed equally to the literature review, writing, discussion and editing. All authors contributed to the article and approved the submitted version.

## Conflict of Interest

The authors declare that the research was conducted in the absence of any commercial or financial relationships that could be construed as a potential conflict of interest.
